# Flow cytometric techniques for isolating and analysing leucocytes

**DOI:** 10.1186/1476-9255-12-S1-O9

**Published:** 2015-04-16

**Authors:** Adriano G Rossi, Fiona Rossi

**Affiliations:** 1 MRC Centre for Inflammation Research, Queen's Medical Research Institute, University of Edinburgh Medical School, 47 Little France Crescent, Edinburgh, EH16 4TJ, UK; 2 MRC Centre for Regenerative Medicine, Scottish Centre for Regenerative Medicine Edinburgh bioQuarter, The University of Edinburgh, 5 Little France Drive, Edinburgh, EH16 4UU, UK

## 

Flow cytometric analysis and flow cytometric cell sorting (FACS) have revolutionized the understanding of immune cell biology and proved an indispensible tool not just for immunologists but for those interested in inflammatory processes. Right through from the early days of impedance-based flow cytometry counter devices to the first fluorescence-based flow cytometry instruments where side and forward scatter parameters with one or two florescent color instruments to state-of-the-art multicolour flow cytometric and mass cytometry with over 38-antibody panels, we are discovering new cell types and multiple immune cell phenotypes that exhibit a diverse array of functions. Today most laboratories use flow cytometry as a powerful technique to support their fundamental biological and medical research. Our own work has used flow cytometry to successfully measure mammalian (e.g., human and mouse) leucocyte function (e.g., cell polarization, reactive oxygen species release, shedding of CD62L, upregulation of CD11b, etc) and apoptosis (shedding of CD16 from neutrophils and fluorescently labeled annexin V binding to apoptotic cell surfaces, DNA hyplodiploid peak assessment of permeabilised cells, macrophage efferocytosis (phagocytosis of apoptotic cells and to sort highly pure non-perturbed neutrophils from autofluorescent eosinophils [1-3]. Furthermore, flow cytometry has been crucial in the identification, characterization and phenotypic analysis of inflammatory leucocytes isolated from healthy volunteers and patients [4] and from cells isolated from various inflammatory sites in mouse models of inflammation [5]. Recent advances in flow cytometry (especially in imaging flow cytometry) will undoubtedly provide further insights and new information in the already complex field of leucocyte biology.
